# Preclinical Activity of Eltrombopag (SB-497115), an Oral, Nonpeptide Thrombopoietin Receptor Agonist

**DOI:** 10.1634/stemcells.2008-0366

**Published:** 2009-02

**Authors:** Connie L Erickson-Miller, Evelyne Delorme, Shin-Shay Tian, Christopher B Hopson, Amy J Landis, Elizabeth I Valoret, Teresa S Sellers, Jon Rosen, Stephen G Miller, Juan I Luengo, Kevin J Duffy, Julian M Jenkins

**Affiliations:** a GlaxoSmithKline, CollegevillePennsylvania, USA; b Ligand PharmaceuticalsLa Jolla, California, USA; c GlaxoSmithKline, King of PrussiaPennsylvania, USA

**Keywords:** Thrombopoietin receptor agonist, Megakaryocyte, Differentiation, Thrombocytopenia

## Abstract

Eltrombopag is a first-in-class, orally bioavailable, small-molecule, nonpeptide agonist of the thrombopoietin receptor (TpoR), which is being developed as a treatment for thrombocytopenia of various etiologies. In vitro studies have demonstrated that the activity of eltrombopag is dependent on expression of TpoR, which activates the signaling transducers and activators of transcription (STAT) and mitogen-activated protein kinase signal transduction pathways. The objective of this preclinical study is to determine if eltrombopag interacts selectively with the TpoR to facilitate megakaryocyte differentiation in platelets. Functional thrombopoietic activity was demonstrated by the proliferation and differentiation of primary human CD34^+^ bone marrow cells into CD41^+^ megakaryocytes. Measurements in platelets in several species indicated that eltrombopag specifically activates only the human and chimpanzee STAT pathways. The in vivo activity of eltrombopag was demonstrated by an increase of up to 100% in platelet numbers when administered orally (10 mg/kg per day for 5 days) to chimpanzees. In conclusion, eltrombopag interacts selectively with the TpoR without competing with Tpo, leading to the increased proliferation and differentiation of human bone marrow progenitor cells into megakaryocytes and increased platelet production. These results suggest that eltrombopag and Tpo may be able to act additively to increase platelet production.

## INTRODUCTION

Thrombocytopenia is a condition of an unusually low level of platelets in the blood and results from an imbalance between the production and destruction of platelets. Thrombocytopenia is associated with several medical disorders, including aplastic anemia [1], myelodysplasia [1], and idiopathic thrombocytopenic purpura (ITP) [2]. Clinically significant thrombocytopenia can occur as a consequence of myelosuppressive or myeloablative chemotherapy or radiotherapy [3]. Thrombocytopenia can also be associated with severe chronic liver disease due to the reduced production of the endogenous thrombopoietic growth factor, thrombopoietin (Tpo), and/or the increased sequestration of platelets [4]. In patients infected with the hepatitis C virus (HCV), thrombocytopenia may occur due to the myelosuppressive effects of the virus on the bone marrow [5].

A key concept in treating thrombocytopenia is to eliminate the underlying problem, which may include targeting the cause of the increased platelet destruction (i.e., immunosuppressive agents or other drugs that may cause thrombocytopenia) or increasing platelet counts by stimulating the production of new platelets. Platelet production initially originates from megakaryocyte precursor cells in the bone marrow. Proliferation and differentiation in the megakaryocytic pathway is predominantly controlled by Tpo, a cytokine that is constitutively produced, and primarily made by the liver [6,7]. The binding of Tpo to the thrombopoietin receptor (TpoR) on cells in the megakaryocyte pathway triggers the activation of the cytoplasmic tyrosine kinases Janus kinase (JAK)2 and tyrosine kinase 2, which in turn activate signal transducers and activators of transcription five (STAT)5, phosphoinositide-3 kinase, and Ras-mitogen-activated protein kinase (MAPK) [8–10]. The subsequent changes in gene expression in precursor cells promote differentiation along the megakaryocytic lineage and have an antiapoptotic effect, which ultimately leads to platelet development and release.

Following the purification of Tpo in the mid-1990s, recombinant human Tpo (rhTpo) and a similar protein, megakaryocyte growth and development factor, were extensively tested for their ability to overcome thrombocytopenia, and were shown to significantly increase circulating platelet levels in mice, primates, and humans [7,11–15]. However, because of the induction of immunogenicity [7,16], megakaryocyte growth and development factor is no longer being tested in clinical trials. Nonpeptide, small-molecule TpoR agonists are an attractive alternative to protein therapeutics because they are more likely to be orally bioavailable and less likely to be immunogenic.

In this report, we present preclinical results for eltrombopag (SB-497115, Promacta®/Revolade™; GlaxoSmithKline, Research Triangle Park, NC, http://www.gsk.com, and Ligand Pharmaceuticals, Inc., San Diego, CA, http://www.ligand.com), an orally bioavailable, small-molecule, nonpeptide TpoR agonist. We show that eltrombopag interacts specifically with the TpoR without competing with Tpo, thereby activating intracellular signal transduction pathways additively with endogenous Tpo, leading to the increased proliferation and differentiation of human bone marrow progenitor cells into megakaryocytes, and ultimately, increased platelet production.

## MATERIALS AND METHODS

### Cytokines and Cell Lines

rhTpo, recombinant human stem cell factor (rhSCF), and recombinant murine interleukin-3 (rmIL-3) were obtained from R&D Systems, Inc. (Minneapolis, MN, http://www.rndsystems.com). Recombinant human erythropoietin and G-CSF were obtained from Amgen, Inc. (Thousand Oaks, CA, http://www.amgen.com). The BAF3/hTpoR and 32D-mpl cell lines were made by stable transfection of rhTpoR (c-mpl) and STAT-inducible reporter interferon regulatory factor one (IRF-1; BAF3/hTpoR cells) or the megakaryocyte-specific promoter glycoprotein (gp)IIb (32D-mpl cells) coupled to the luciferase reporter gene, as previously described [17]. These two TpoR-transfected cell lines are dependent on rmIL-3 or rhTpo for growth. The N2C-Tpo cell line, derived by culture of a megakaryoblastic cell line in rhTpo for 10 weeks, was generously provided by Dr. Camille Abboud of the University of Rochester Medical Center (Rochester, NY).

### Luciferase Reporter Gene Assay

BAF3/hTpoR or 32D-mpl cells were washed and starved of rmIL-3 or rhTpo overnight prior to treatment. Starved parental BAF3 cells (1 × 10^5^ cells/ml) in Iscove's modified Dulbecco's medium (IMDM)/0.5% fetal bovine serum (FBS) and 30 μM ZnCl_2_ were treated with eltrombopag (0.002–50 μM) or rhTpo (100 ng/ml) at 37°C, 5% CO_2_, for 4 hours. Cells were lysed in 100 μl lysis buffer (25 mM tris, 15% glycerol, 2% 3-[(3-cholamidopropyl)dimethylammonio]-1-propanesulfonic acid, 1% lecithin, 1% bovine serum albumin, 4 mM EGTA, 8 mM MgCl_2_, 10 mM dithiothreitol, and 0.4 mM phenylmethylsulfonyl fluoride, pH 7.8) for 15 minutes and added to a 96-well plate (30 μl per well). Promega Steady Glow (100 μl; Madison, WI, http://www.promega.com) was added to each well immediately before the plates were read using a chemiluminometer (Model ML1000; Dynatech Laboratories Inc., Chantilly, VA, http://dynatechlaboratories.com). Luciferase assay data are presented as the mean and standard error of quadruplicate wells.

### Proliferation Assays

Bromodeoxyuridine (BrdU) proliferation assays were conducted using cytokine-starved BAF3/hTpoR cells plated in 96-well plates at 4 × 10^4^ cells/well. rhTpo (100 ng/ml) or eltrombopag (0.0001–10 μM) was added to triplicate wells and the cells were incubated in 5% CO_2_ at 37°C. BAF3/hTpoR cells were grown for 44 hours, labeled with BrdU, and returned to the incubator for four more hours. The plates were developed using a BrdU cell proliferation kit (Roche Diagnostics, Indianapolis, IN, http://www.roche-diagnostics.us) and were read on an enzyme-linked immunosorbent assay plate reader at 380 nm.

Thymidine incorporation assays were conducted using cytokine-starved N2C-Tpo cells plated in 96-well plates (1.4 × 10^5^ cells/ml final concentration), grown in a white view plate, and exposed to eltrombopag (0.003–3 μM) and/or rhTpo (1–100 ng/ml) for 72 hours at 37°C. Tritiated thymidine (1 μCi/well) was added for the final 4 hours of incubation. Cells were harvested onto glass fiber filter mats and read on a Wallac 1,450 Microbeta scintillation counter (PerkinElmer, Inc., Waltham, MA, http://www.perkinelmer.com).

### Caspase-3 and Caspase-7 Assays

The Caspase-Glo 3/7 assay (Promega) is a luminescent assay that measures caspase-3 and caspase-7 activity. The addition of the Caspase-Glo reagent results in cell lysis, followed by caspase cleavage of the substrate and generation of a luminescent signal; the amount of luminescence is proportional to the amount of caspase present. Cytokine-starved N2C-Tpo cells (1.4 × 10^5^ cells/ml final concentration) were grown in a white view plate and exposed to eltrombopag (0.003–3 μM) and/or rhTpo (1–100 ng/ml) for 72 hours at 37°C. Caspase-Glo (100 μl) was added, and cells were incubated for 90 minutes at room temperature. Luminescence was measured using the Envision plate reader (PerkinElmer, Inc.).

### Western Blot Analysis of STAT5 and MAPK

N2C-Tpo cells (1 × 10^6^) were starved of rhTpo overnight in IMDM-containing glutamine and 0.5% FBS. Cells were treated for up to 120 minutes with eltrombopag (30 μM) or rhTpo (75 ng/ml), pelleted by centrifugation, and placed on dry ice. Lysates were prepared, proteins were separated by gel electrophoresis, and immunoblotting was performed with antibodies to phospho-STAT5 and p44/42 MAPK, as described previously [17].

CD34^+^ cells (5 × 10^5^ cells/ml) were cultured in StemSpan (Stem Cell Technologies, Vancouver, Canada, http://www.stemcell.com) and rhTpo (100 ng/ml). After 10 days, CD41^+^ megakaryocytes comprised 75% of the cells. These cells were washed and starved of rhTpo for 6 hours, treated with rhTpo (100 ng/ml) or eltrombopag (0, 1, 3 or 10 μM) for 30 minutes, and made into lysates. Lysates were applied to a 12% Tris-Bis gel (50 μg per lane), and anti-phopsho-STAT5 antibody (cat#AB3800; Millipore, Billerica, MA, http://www.millipore.com) was used to detect activation of STAT5.

### Human Marrow Differentiation Assay

Human blood and bone marrow samples were obtained from healthy donors with informed consent under an institutional review board-approved protocol. CD34^+^ cells were purified from the light density fraction of normal human bone marrow by Miltenyi (Auburn, CAhttp://www.miltenyibiotec.com) immunomagnetic bead separation as previously described [17]. CD34^+^ cells were plated at 1 × 10^5^ cells/ml in IMDM with 20% FBS and 100 ng/ml rhSCF. Eltrombopag (0.003–3.0 μM) or rhTpo (100 ng/ml) was added at 10% final volume and the cells were incubated for 10 days at 37°C in 5% CO_2_ and 5% O_2_. Cells were stained with fluorescein isothiocyanate-conjugated anti-CD41a (gpIIb), isotype control antibody, or phosphate-buffered saline as an autofluorescence control. Flow cytometric analysis was performed on a Becton-Dickinson FACScan (BD Immunocytometry Systems, San Jose, CA, http://www.bdbiosciences.com). Gates for CD41^+^ cells were based on the negative control (i.e., SCF-treated cultures). Data are represented as a percentage of Tpo_max_ (Tpo_max_ = [% CD41_sample_ − % CD41_SCF_]/[% CD41_Tpo_ − % CD41_SCF_]).

### Electrophoretic Mobility Shift Assay

Human, chimpanzee, cynomolgus, and murine blood samples were obtained using the appropriate Institutional Animal Care and Use Committee-approved protocols. Platelets were incubated with eltrombopag (0.3–30 μM) or rhTpo (100 ng/ml) for 20 minutes at 37°C. Lysates and the IRF-1 DNA probe were prepared as previously described [17]. ^32^P-IRF-1 was incubated with the platelet lysate at room temperature for 20 minutes and loaded onto a 5% acrylamide gel. Results were analyzed after visualization by autoradiography.

### Chimpanzee Study Protocol

Chimpanzee data were collected under an Institutional Animal Care and Use Committee-approved protocol at the Southwest Foundation for Biomedical Research (San Antonio, TX, http://www.sfbr.org). Female chimpanzees (approximately 7–8 years of age) were given either eltrombopag (10 mg/kg) in aqueous 2% hydroxypropyl methylcellulose with 0.2% sodium lauryl sulfate vehicle or vehicle alone by oral gavage at a dose volume of 1 ml/kg. Chimpanzees were given five daily doses of vehicle alone (*n* = 2) or eltrombopag (*n* = 3). Platelet counts and reticulated platelet counts were performed prior to, during, and following the treatment regimen. At the end of the study, all chimpanzees were returned to the stock colony.

## RESULTS

### Eltrombopag Molecular Structure and Characterization

**Figure 1:**
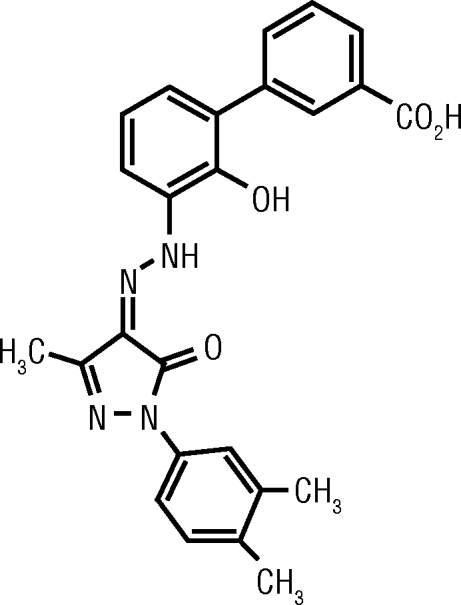
Structure of eltrombopag, an orally bioavailable thrombopoietin receptor agonist.

**Figure 2:**
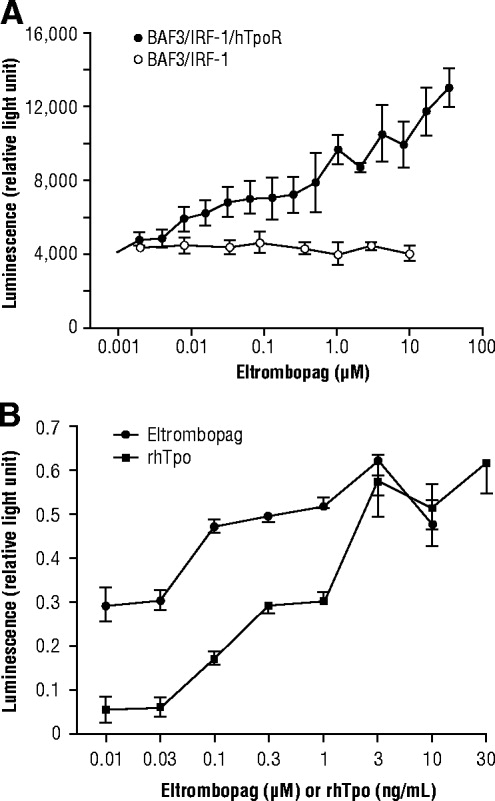
Cell-based STAT activation. **(A):** Representative gene activity of eltrombopag on BAF3/interferon regulatory factor (IRF)-1 cells transfected with and without the thrombopoietin receptor (TpoR). (**B**): Gene activity of eltrombopag and recombinant human thrombopoietin (rhTpo) on 32D-mpl cells transfected with the megakaryocyte-specific glycoprotein IIb promoter. Reprinted from Duffy KJ, Erickson-Miller CL. The discovery of eltrombopag, an orally bioavailable TpoR agonist. In: Metcalf BW, Dillon S, eds. Target Validation in Drug Discovery. Burlington, MA: Academic Press, 2007:241–254, with permission.

Eltrombopag is a member of the biarylhydrazone class of compounds, with an empirical formula of C_25_H_22_N_4_O_4_ and a molecular weight of approximately 442 D (Fig. 1). Eltrombopag demonstrated a half maximal effective concentration (EC_50_) of 0.27 μM in murine BAF3 cells transfected with the luciferase reporter gene under direction of the STAT-activated IRF-1 promoter and human TpoR (BAF3/IRF-1/hTpoR) (Fig. 2 A). Because eltrombopag is specific to the human and chimpanzee TpoR, the parental murine BAF3 cell line, which was not transfected with hTpoR (BAF3/IRF-1), was not activated by eltrombopag (Fig. 2A). Eltrombopag and rhTpo both demonstrated activity in hTpoR-transfected 32D-mpl cells using the reporter assay with luciferase under the direction of the megakaryocyte-specific promoter gpIIb (Fig. 2B).

### Specificity of Eltrombopag Binding

To determine whether the effect of eltrombopag was specific for the activation of the TpoR, cell lines that are dependent on other cytokines for growth were tested. In a variety of different assays, eltrombopag showed no activity in cells expressing receptors for hematopoietic growth factors that are activated by STAT, including Epo, G-CSF, interferon (IFN)-α, IFN-γ, and IL-3 (Table 1).

**Table 1:** Selectivity of eltrombopag for TpoR-expressing cells

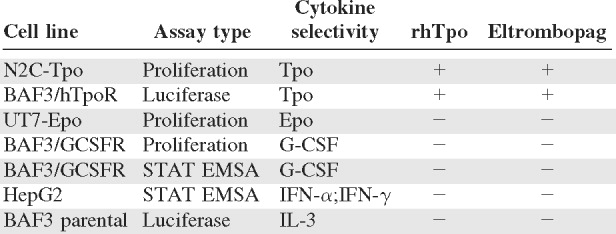

Abbreviations: EMSA, electrophoretic mobility shift assay; Epo, erythropoietin; IFN, interferon; IL-3, interleukin 3; rhTpo, recombinant human thrombopoietin; STAT, signal transducers and activators of transcription; Tpo, thrombopoietin; TpoR, thrombopoietin receptor.

**Figure 3:**
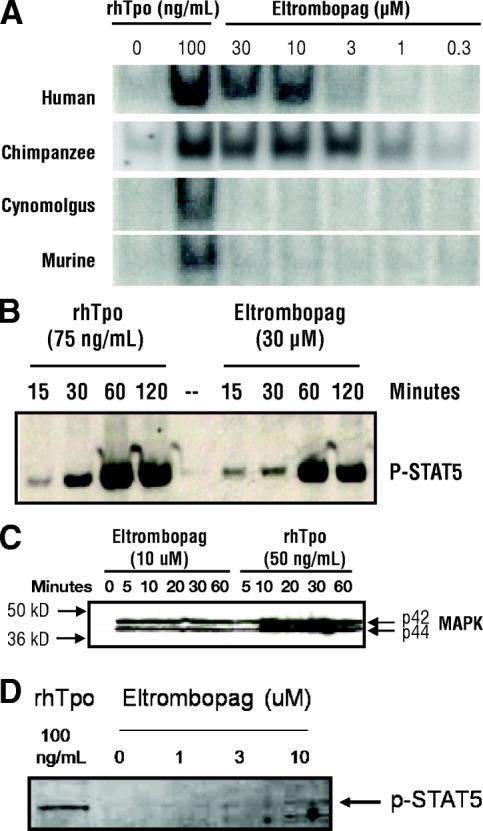
Signal transduction induced by eltrombopag. **(A):** Eltrombopag demonstrated activity on human and chimpanzee (but not other species) platelets by signaling transducers and activators of transcription (STAT) electrophoretic mobility shift assay. Similar kinetics of **(B)** phospho-STAT5 and **(C)** mitogen-activated protein kinase (MAPK) expression were detected by Western blot of eltrombopag- or recombinant human thrombopoietin (rhTpo)-treated N2C-Tpo cell lysates. **(D):** STAT5 activation was also detected in megakaryocytes derived from CD34^+^ cells. **(A, B):** Reprinted from Duffy KJ, Erickson-Miller CL. The discovery of eltrombopag, an orally bioavailable TpoR agonist. In: Metcalf BW, Dillon S, eds. Target Validation in Drug Discovery. Burlington, MA: Academic Press, 2007:241–254, with permission.

The pharmacophore defined by the biarylhydrazone class of compounds [18–20] has a distinct species specificity. In electrophoretic mobility shift assays measuring STAT activation induced by such compounds, including eltrombopag, activation was detected only in human or chimpanzee platelets (Fig. 3 A). A single amino acid difference in the transmembrane domain (histidine 499 of human TpoR) [21] is hypothesized to be responsible for this species specificity. Studies of the mechanism of action of eltrombopag and compounds bearing a similar pharmacophore suggest that eltrombopag activates the receptor by association with metal ions (i.e., Zn^2+^) and specific amino acids within the transmembrane and juxtamembrane domains of the TpoR [17,22,23].

### Activation of TpoR Signaling Pathways

As measured by Western blotting, the activation of the TpoR via interaction with eltrombopag results in the induction of intracellular signaling pathways similar to those induced by rhTpo, but with less intensity. N2C-Tpo cells are a Tpo-dependent human cell line that endogenously expresses the TpoR. Incubation of N2C-Tpo cells with eltrombopag at a 30-μM concentration resulted in activation of STAT5, as detected with an antiphospho-STAT5 antibody on Western blots. Maximum signal intensity was observed at 60 minutes after treatment with eltrombopag (Fig. 3B). The time course of activation by eltrombopag was similar to that achieved by 75 ng/ml rhTpo. Eltrombopag (10 μM) also activated p42/44 MAPK with similar kinetics to rhTpo (50 ng/ml) (Fig. 3C). The detection of STAT5 phosphorylation at 30 minutes after treatment of megakaryocytes derived from CD34^+^ cells with rhTpo or eltrombopag demonstrated a detectable activation of STAT5 by rhTpo. As in the N2C-Tpo cells, the activation of STAT5 by eltrombopag in these megakaryocytes was dose-dependent, but at a much lower level than the signal induced by rhTpo (Fig. 3D).

### Stimulation of Proliferation and Differentiation in Mammalian Cells and Cell Lines

**Figure 4:**
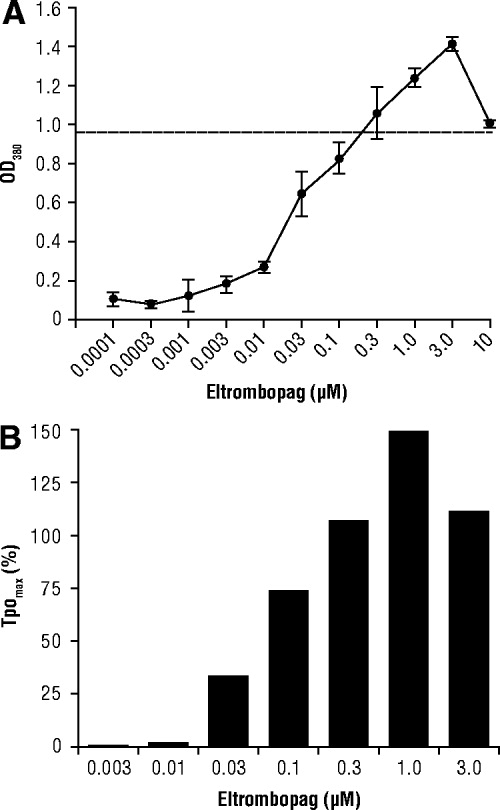
Proliferation and differentiation induced by eltrombopag. **(A):** Proliferation of BAF3/hTpoR cells induced by eltrombopag after 48 hours of treatment; the dotted line represents the activity of cells treated with 100 ng/ml of recombinant human thrombopoietin. OD, optical density. **(B):** Representative example of megakaryocyte differentiation of CD34^+^ cells after 10 days of eltrombopag treatment; similar results were obtained with six independent marrow samples. Panel **(B)** was reprinted from Duffy KJ, Erickson-Miller CL. The discovery of eltrombopag, an orally bioavailable TpoR agonist. In: Metcalf BW, Dillon S, eds. Target Validation in Drug Discovery. Burlington, MA: Academic Press, 2007:241–254, with permission.

In a BrdU assay conducted in BAF3/hTpoR cells, eltrombopag stimulated proliferation after a 2-day incubation with an EC_50_ of 0.03 μM (Fig. 4A).

In addition to a proliferative effect on cells of the megakaryocytic lineage, eltrombopag also induces differentiation of hematopoietic stem cells into committed megakaryocyte progenitor cells. Assessment of megakaryocyte maturation can be determined by measuring the appearance of the megakaryocyte-specific marker glycoprotein CD41 on human CD34^+^ cells obtained from human bone marrow samples. The data were calculated as a percentage of the maximal number of cells responding to the maximally efficacious dose of rhTpo (i.e., 100 ng/ml). Eltrombopag increased the differentiation of bone marrow CD34^+^ cells into CD41^+^ megakaryocytes in a dose-dependent manner with an EC_50_ of 0.1 μM (Fig. 4B).

**Figure 5:**
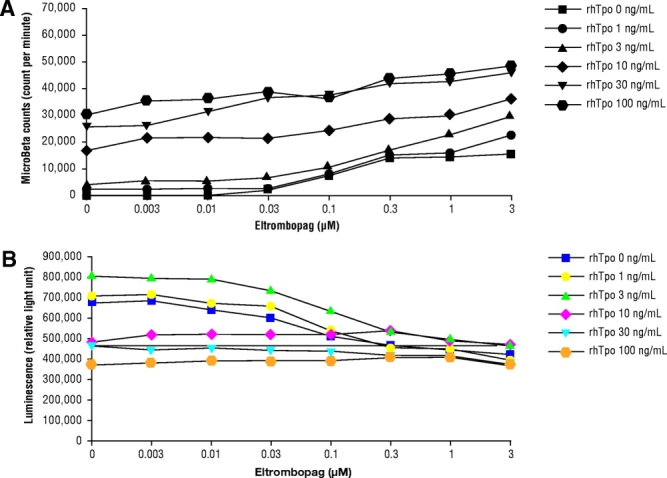
Additive effects of eltrombopag and rhTpo. **(A):** Proliferation, as measured by thymidine incorporation, of N2C-Tpo cells by eltrombopag (0.003–3 μM) in combination with recombinant human thrombopoietin (rhTpo; 1–100 ng/ml). **(B):** Activation of caspase-3 and caspase-7 by eltrombopag (0–3 μM) in combination with rhTpo (0–100 ng/ml) in N2C-Tpo cells.

Thymidine incorporation assays in N2C-Tpo cells were utilized to determine the effect of treatment with eltrombopag in combination with rhTpo. The results demonstrate that eltrombopag and Tpo are not antagonistic with each other; rather, there appears to be an additive effect when eltrombopag is added to suboptimal amounts of rhTpo (Fig. 5 A). The additive effect of eltrombopag also occurs in the presence of rhTpo at a concentration that causes a plateau in cell proliferation rates (i.e., 100 ng/ml).

### Caspase-3 and Caspase-7 Assays in Cells Incubated with Eltrombopag and rhTpo

In an assay measuring the activation of caspase-3 and caspase-7, treating N2C-Tpo cells with rhTpo at concentrations of 0, 1, and 3 ng/ml, and with eltrombopag at concentrations of 0–0.03 μM, demonstrated comparable caspase activity to untreated cells, indicating that these concentrations of rhTpo and eltrombopag were insufficient to prevent apoptosis of N2C-Tpo cells (Fig. 5B). The antiapoptotic effect evidenced by lowered caspase cleavage activity occurred at concentrations of eltrombopag >0.03 μM. Increasing concentrations of eltrombopag demonstrated additional antiapoptotic effects in these cells. Cells incubated with 10, 30, or 100 ng/ml rhTpo also demonstrated decreased caspase activation and an antiapoptotic effect on these cells. Thus, both Tpo and eltrombopag are capable of preventing apoptosis in Tpo-dependent cells and could substitute for each other at suboptimal levels of each treatment.

### Repeat Dose Study in Chimpanzees

**Figure 6:**
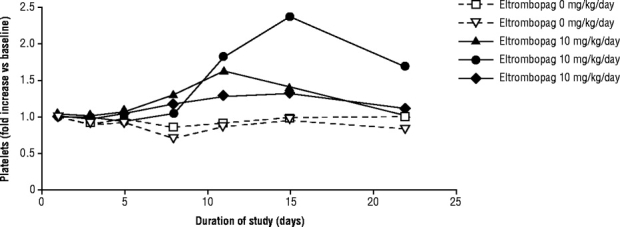
Increase in platelet counts in chimpanzees treated orally with eltrombopag for 5 days at 10 mg/kg per day (solid lines; *n* = 3) or vehicle (dotted lines, *n* = 2). Data are expressed as the fold increase of each animal over its baseline platelet count. Reprinted from Duffy KJ, Erickson-Miller CL. The discovery of eltrombopag, an orally bioavailable TpoR agonist. In: Metcalf BW, Dillon S, eds. Target Validation in Drug Discovery. Burlington, MA: Academic Press, 2007:241–254, with permission.

Given the distinct species specificity for STAT activation exhibited by eltrombopag in human and chimpanzee platelets, a 5-day, repeat-dose safety and pharmacology study was conducted in five female chimpanzees. Chimpanzees were administered either vehicle alone (*n* = 2) or eltrombopag in vehicle (*n* = 3; 10 mg/kg per day) by oral gavage. Administration of eltrombopag was well tolerated following repeat oral doses. There were no adverse effects on hematology, coagulation, or clinical chemistry parameters. In chimpanzees treated with eltrombopag, platelet counts were increased over twofold approximately 1 week after the last dose for one chimpanzee and approximately 1.5-fold for the other two chimpanzees. Platelet counts returned to near baseline values approximately 2 weeks after peak values were reached (Fig. 6).

Although only limited pharmacokinetic sampling in three chimpanzees was performed, the data suggest that the pharmacodynamic signal of a change in platelet count from baseline for eltrombopag in the chimpanzees was detected at minimum concentration (C_min_), C_max_, and area under the curve (AUC) values of approximately 0.107 μg/ml, 0.525 μg/ml, and 12.1 μg h/ml, respectively.

## DISCUSSION

Eltrombopag represents the first in a class of orally bioavailable, nonpeptide TpoR agonists. In these preclinical studies, we have shown that eltrombopag activates TpoR with kinetics similar to rhTpo. Eltrombopag has high potency in in vitro assays for activation of the STAT and MAPK signaling pathways, and proliferation of Tpo-dependent cell lines. In addition, eltrombopag induced the differentiation of bone marrow precursor cells, the ultimate target cell of patients with thrombocytopenia, with EC_50_ values in the range of 30–300 nM. These activities of eltrombopag were shown to be dependent on the expression of the TpoR. Cells expressing other growth factor receptors, even those acting through the JAK/STAT signaling pathway, were not activated by eltrombopag, suggesting that the activation of JAK/STAT by eltrombopag is due to the specific activation of the TpoR. In addition, eltrombopag and Tpo do not bind to the same site on the TpoR, which prevents competitive binding and allows eltrombopag and Tpo to have additive cell-signaling effects. Differences in some aspects of the signaling pathway between eltrombopag and rhTpo (i.e., activation of AKT) have been detected in platelets [24].

Because eltrombopag is specific for humans and chimpanzees, preclinical in vivo assessments of pharmacologic activity were limited to chimpanzees. Oral administration of 10 mg/kg of eltrombopag per day to chimpanzees was shown to increase platelet counts after 5 days of administration, which is similar to the kinetics of rhTpo. These in vivo data, combined with the EC_50_ in vitro differentiation results, provided a rationale for examining the target effect exposure in healthy subjects in a phase I clinical study [25]. Oral administration of eltrombopag leading to a steady-state minimal concentration of >225 ng/ml and an AUC of 30 μg h/ml was targeted as the minimally effective pharmacokinetic activity for human studies.

The administration of recombinant Tpo to rodents, primates, and humans has been shown to significantly increase circulating platelet levels [11–15,26,27]. However, attempts to develop an rhTpo have been unsuccessful, due in part to the development of neutralizing antibodies to endogenous Tpo in some patients who received megakaryocyte growth and development factor [16]. Nonpeptide, small-molecule TpoR agonists like eltrombopag are less likely to induce immunogenicity and may also be conveniently administered orally.

The development of targeted TpoR agonists has implications for improving therapy for patients suffering from diseases associated with low platelet counts. For example, ITP treatment has focused on reducing autoimmune-induced platelet destruction through the use of immunosuppressive or immunomodulatory therapies, such as corticosteroids and injectable pooled immunoglobulin preparations, or even splenectomy. Data have emerged supporting the role of inadequate platelet production in ITP, and eltrombopag has been successful in boosting platelet counts in ITP patients [28]. Thrombopoietin receptor agonists may also prove to have clinical benefit in other conditions in which thrombocytopenia can occur, such as myelodysplastic syndrome [29] and HCV [30]. In addition to evaluating the clinical benefit of eltrombopag, the effects of eltrombopag and other TpoR agonists on signaling, proliferation, differentiation, and receptor internalization, among others, suggest many areas of future preclinical research opportunity.

## CONCLUSION

The Tpo mimetic properties of eltrombopag have been demonstrated in vitro and in animal studies, and have provided the foundation for the clinical development of eltrombopag in a broad range of thrombocytopenia-associated conditions. Because eltrombopag does not compete with Tpo for binding to the TpoR, eltrombopag and endogenous Tpo may be able to work together to activate platelet production. Collectively, these data suggest that oral eltrombopag has great potential for combating thrombocytopenia of various etiologies.
